# Sclerosing Polycystic Adenosis of the Retromolar Pad Area: A Case Report

**DOI:** 10.1155/2014/982432

**Published:** 2014-03-03

**Authors:** Sepideh Mokhtari, Saede Atarbashi Moghadam, Abbas Mirafsharieh

**Affiliations:** ^1^Department of Oral and Maxillofacial Pathology, Dental School of Shahid Beheshti University of Medical Sciences, Tehran 19857-17443, Iran; ^2^Modarres Hospital, Department of Pathology, Shahid Beheshti University of Medical Sciences, Tehran 19857-17443, Iran

## Abstract

Sclerosing polycystic adenosis is a rare pathological lesion that affects salivary glands. The majority of cases involve the parotid and its occurrence in minor glands is exceedingly rare. Here, we report the first case of this lesion in the retromolar pad area and discuss its histological features and immunohistochemical reactivity with **α**SMA and Ki67 markers. A review of the literature on its immunohistochemical profile is also provided. Sclerosing polycystic adenosis has a diverse histomorphology and should be differentiated from other more important pathologic lesions.

## 1. Introduction

To the best of our knowledge, 54 cases of sclerosing polycystic adenosis (SPA) of salivary glands have been reported. SPA characteristically arises in the major glands, and the majority of cases involve the parotid [1]. Some cases have also been reported in minor salivary glands of mucobuccal fold, hard palate, floor of mouth, and buccal mucosa [2, 3]. SPA has been reported in a wide age range from childhood to the eighth decade of life [1]. Here, we report the first case of SPA in the retromolar pad area.

## 2. Case Report 

**Figure 1:**
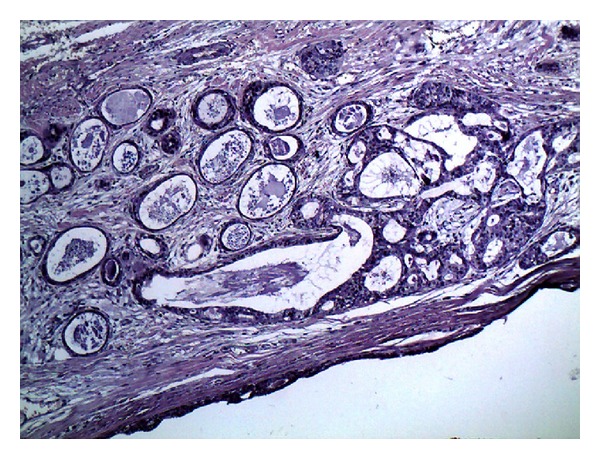
Large cystic spaces and cribriform structures were present throughout the lesion (×100).

**Figure 2:**
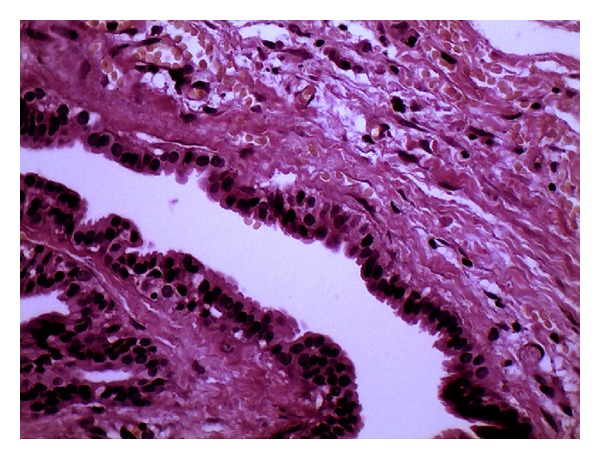
Apocrine metaplasia was evident throughout the lesion (×400).

**Figure 3:**
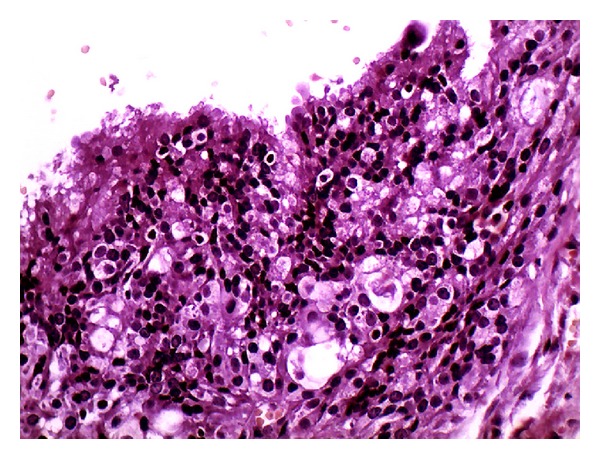
Mucous cells were frequently seen (×400).

A 60-year-old male presented with swelling in his retromolar pad area with two months' duration. There was no tenderness or ulceration. Excisional biopsy of the lesion was performed and a well-circumscribed soft tissue lesion was excised. Histopathologic examination showed lobules of hyalinized connective tissue with epithelial components of ductal and acinar differentiation. Ductal structures formed variably sized cysts or they were packed as small ducts similar to the sclerosing adenosis of the breast. Ducts were lined by flattened to cuboidal epithelial cells and some cells had apocrine metaplasia. Mucous cells were frequently seen (Figures 1, 2, and 3). Periductal fibrosis with lamellar architecture was a common feature. Occasional hyaline globules were also present. Epithelial hyperplasia of ductal structures, formed solid nests, cribriform structures and intraductal anastomosing bridges. Few chronic inflammatory cells were infiltrated throughout the lesion.

**Figure 4:**
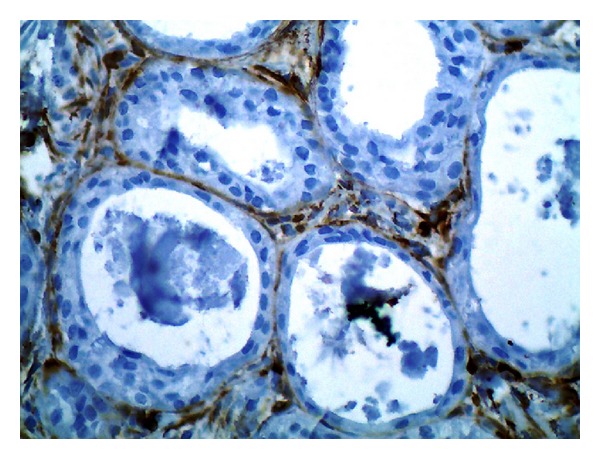
Immunohistochemical examination with *α*SMA confirmed the presence of a peripheral myoepithelial layer around all ductal structures (×400).

**Figure 5:**
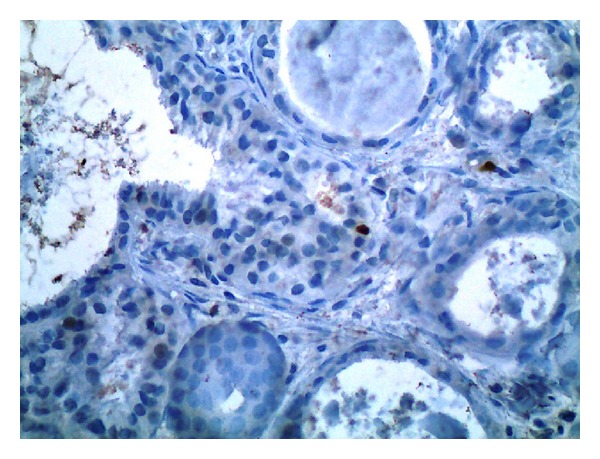
Less than 1% of lesional cells were immunoreactive for Ki-67 antibody (×400).

Immunohistochemical staining for *α*SMA and Ki-67 was performed. Myoepithelial cells, surrounding ductal elements, demonstrated immunoreactivity for *α*SMA (Figure 4). Immunohistochemical examination with Ki-67 revealed less than 1% positivity in lesional cells (Figure 5). The proliferative cells were present within ductal elements of cribriform structures, which explained transluminal duct hyperplasia.

## 3. Discussion

There is a controversy whether SPA is a neoplasm or reactive lesion. Clonal nature of cells has been demonstrated in some cases [4]. Some viruses such as human papilloma virus (HPV) and Epstein-Barr virus (EBV) may also have a role in pathogenesis of salivary gland diseases. One recent study has demonstrated EBV expression in tumor cells supporting the neoplastic nature of SPA and a possible association with Epstein-Barr virus. Interestingly, no immunoreactivity has been observed for HPV [5].

Reports of cytological atypia or dysplasia within some SPA have added to controversies about the nature of this lesion. Atypia may be found within the ductal epithelial cells ranging from mild to severe dysplasia and carcinoma in situ. However, the lobular architecture is always maintained and invasive carcinoma has not been identified in SPA cases [6].

SPA has diverse histological features. This lesion has a strong resemblance to the fibrocystic disease of breast [2]. Sclerosis and marked adenosis of ductal elements were evident in this case, but adenosis of acini was lacking. Sebaceous, squamous, foamy, and vacuolated cells as well as acinar cells with cytoplasmic zymogen granules have been described in this lesion [2]. However, our case was devoid of these features. Gnepp et al. have also reported two cases with lipomatous stroma [7].

Some authors have investigated immunohistochemical staining profile of cells in SPA. A review of previous studies is presented in Table 1. However, more investigations are required to establish the immunohistochemical profile of this lesion.

SPA is treated with conservative surgical excision with tumor-free margins and recurrence is rarely encountered [8].

## Figures and Tables

**Table 1:** A review of immunohistochemical investigations in SPA cases.

Investigators	Markers	Immunohistochemical reactivity
Fulciniti et al. (2010) [9]	Collagen IV	Enhanced lobular architecture
	Cytokeratin 14	Enhanced the ratio of apocrine cells present in the epithelial lining of lobular structures
	Gross cystic disease fluid protein (GCFDP)	Sebaceous cells

Gurgel et al. (2010) [8]	Ki-67	Positive in less than 1% of cells
	CKAE1/AE3, EMA, GCDFP-15	Tubuloacinar elements
	Estrogen, progesterone, and CK 34*β*E12	Negative
	SMA, S100	Myoepithelial layer

Swelam (2010) [5]	S100	Lesional ductal and spindle-shaped cells
	Bcl-2	Strong, diffuse cytoplasmic immunoreactivity in basal cells of neoplastic cells
	Ki-67	Sporadic positivity in Basal cells of neoplastic ductal epithelium
	EBV	Expression in neoplastic S100 positive cells
	HPV-1	Negative

Meer and Altini (2008) [2]	P63	Peripheral layer of cells surrounding acini, ducts, and cystic spaces outlining these structures
	AE1/AE3	In ductal lining cells of tubuloacinar elements
	S100	Ductal cells and spindled myoepithelial cells
	AE1/AE3, CAM5.2, EMA, antimicrobial antibody, BRST-2, S100	Luminal cells

Bharadwaj et al. (2007) [10]	Cytokeratin	In ductal and acinar elements
	SMA, S100	Myoepithelial layer

Skálová et al. (2006) [4]	CKAE1/AE3	Positive in ductal and acinar cells
	EMA, S100, antimitochondrial antibody	Variably positive
	CEA, p53, and HER-2/neu	Negative
	GCDFP-15	Acinar cells with coarse eosinophilic cytoplasm
	Progesterone receptors	Positive in 15% to 20% of epithelial cells
	Estrogen receptors	At least focally in 5% of ductal cells in dysplastic and hyperplastic foci
	SMA, P63, and calponin	Myoepithelial layer

Gnepp et al. (2006) [7]	Calponin, SMA, muscle specific actin, S100	Myoepithelial layer
